# Preparation of Polyphosphazene Hydrogels for Enzyme Immobilization

**DOI:** 10.3390/molecules19079850

**Published:** 2014-07-08

**Authors:** Yue-Cheng Qian, Peng-Cheng Chen, Gui-Jin He, Xiao-Jun Huang, Zhi-Kang Xu

**Affiliations:** MOE Key Laboratory of Macromolecular Synthesis and Functionalization, Department of Polymer Science and Engineering, Zhejiang University, Hangzhou 310027, China; E-Mails: education@zju.edu.cn (Y.-C.Q.); chenpengcheng@zju.edu.cn (P.-C.C.); guikinghe@zju.edu.cn (G.-J.H.); xuzk@zju.edu.cn (Z.-K.X.)

**Keywords:** polyphosphazene hydrogel, swelling, lipase, entrapment, activity

## Abstract

We report on the synthesis and application of a new hydrogel based on a methacrylate substituted polyphosphazene. Through ring-opening polymerization and nucleophilic substitution, poly[bis(methacrylate)phosphazene] (PBMAP) was successfully synthesized from hexachlorocyclotriphosphazene. By adding PBMAP to methacrylic acid solution and then treating with UV light, we could obtain a cross-linked polyphosphazene network, which showed an ultra-high absorbency for distilled water. Lipase from *Candida rugosa* was used as the model lipase for entrapment immobilization in the hydrogel. The influence of methacrylic acid concentration on immobilization efficiency was studied. Results showed that enzyme loading reached a maximum of 24.02 mg/g with an activity retention of 67.25% when the methacrylic acid concentration was 20% (w/w).

## 1. Introduction

Enzymes that display high catalytic efficiency and selectivity under mild conditions are promising catalysts for use in many fields such as fine chemical synthesis, food processing, biosensor fabrication and bioremediation [1,2,3,4]. However, the practical applications of native enzymes suffer severely due to low enzyme stability upon exposure to heat, extreme pH values, and organic solvents. Applications are also limited by separation and reuse difficulties. The protocol of enzyme immobilization can hopefully alleviate these problems and facilitate enzyme usage in continuous production processes, which are favorable for industrial applications [5,6,7,8]. In principle, enzymes are immobilized via four major routes: physical adsorption, chemical binding, entrapment, and cross-linking [9]. Among these routes, entrapment stands out as having the advantage of preventing enzymes from direct contact with the environment, thereby minimizing the effects of gas bubbles, mechanical sheer and adverse solvents and possibly stabilizing multimeric enzymes [10,11,12,13,14]. Moreover, entrapment leads to residual mobility and flexibility of the enzyme after immobilization [15,16]. However, every coin has two sides. Apart from the advantages mentioned above, the entrapment route does suffer from mass transfer limitations and sometimes a lack of potential to stabilize enzyme via multipoint covalent attachment [17,18].

In literature, various hydrogels have been used as supports for enzyme entrapment. A hydrogel is a cross-linked polymeric network that is three dimensional, swellable, and yet, insoluble. The nature of cross-linking in the hydrogel network, either physical or chemical, can influence the three-dimensional network structure and properties such as strength, degradation rate, and swelling ratios [19,20]. Because of their biocompatibility and high water absorbability, hydrogels are advantageous for many biological applications, including controlled drug release, cell delivery, artificial organs, biosensors, contact lenses, and enzyme immobilization [21,22]. Enzymes have been entrapped in various hydrogels such as polysaccharides, gelatin, and mostly, carbon-based polymers [23,24,25,26,27].

Polyphosphazenes are hybrid inorganic-organic polymers with alternating phosphorus-nitrogen elements in the backbone and organic side groups attached [28]. Foundation work with polyphosphazene hydrogels and immobilized biomolecules has been conducted by Allcock *et al*. [29,30,31,32,33,34,35]. The use of polyphosphazene hydrogels for enzyme immobilization has some attractive aspects and advantages over typically used carbon-based polymers such as difunctionalized poly(ethylene glycol) (PEG). Firstly, each repeating unit of a polyphosphazene can bear two functional side groups; thus, a high loading of cross-linkable moieties may be attained [36,37]. In contrast, PEG has only two cross-linkable sites per polymer chain. Secondly, the polyphosphazene backbone is transparent at mid- to long-wavelength UV radiation, unlike many carbon-based polymers that tend to absorb UV light in this range. Thus, the probability of side reactions by chain cleavage is reduced. Thirdly, polyphosphazenes bear greater synthetic flexibility, and thus, more control over the chemical and physical properties of a material is possible [38]. This allows the design of many variations of the polymer for specific applications.

In this work, poly[bis(methacrylate)phosphazene] (PBMAP) hydrogels were prepared and used for enzyme entrapment. Using classical nucleophilic substitution, methacrylate groups can be easily incorporated as the side chains of a polyphosphazene. Hydrogels were prepared by cross-linking PBMAP with methacrylic acid under UV light. Lipase from *Candida rugosa* was used as the model lipase for entrapment immobilization. This work hopes to offer a convenient pathway for fabricating a highly efficient enzyme immobilization system and thereby broaden the application of polyphosphazenes.

## 2. Results and Discussion

### 2.1. Synthesis and Characterization of PBMAP

**Figure 1:**
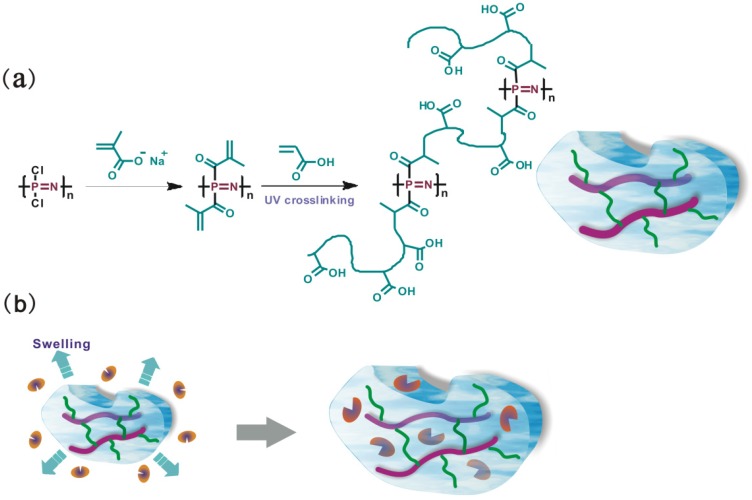
Schematic representation of (**a**) PBMAP hydrogel fabrication and (**b**) enzyme entrapment.

**Figure 2:**
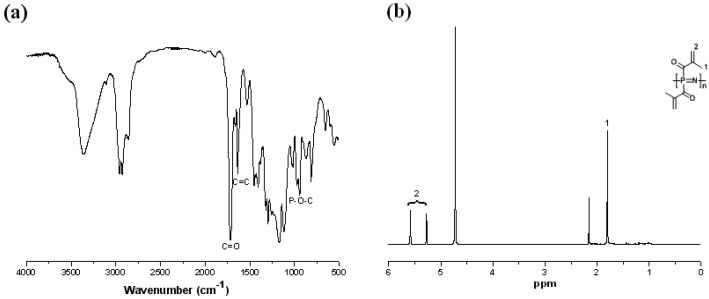
(**a**) FT-IR and (**b**) ^1^H-NMR spectra of PBMAP.

PBMAP was synthesized by the nucleophilic substitution of sodium methacrylate (Figure 1a). Generally, the substitution of chlorine atoms by methacrylate groups is more difficult than traditional alkoxy substitutions because of the lower reactivity of carboxylates as nucleophiles compared to alkoxides [22]. To optimize the macromolecular substitution, the temperature was raised to ~ 35 to 40 °C during the reaction. The structure of the resultant polymer was confirmed by FT-IR and ^1^H-NMR spectra. In the FT-IR spectrum of PBMAP (Figure 2a), the bands corresponding to C=O (1716 cm^−1^), C=C (1635 cm^−1^) and P−O−C (976 cm^−1^) stretching demonstrated that alkyne side groups were successfully incorporated into the polyphosphazene. In addition, bands at 1238 cm^−1^ and 1190 cm^−1 ^confirmed the presence of P−N and N=P groups, respectively. The ^1^H-NMR confirmed that the chlorine atoms were substituted by sodium methacrylate (Figure 2b). The signals at δ = 5.25 and 5.58 correspond to CH_2_=C groups while the signal at δ = 1.83 corresponds to −CH_3_ groups. Moreover, because of the presence of the reactive vinyl group, the polymer underwent some extent of cross-linking during the purification process, which was suggested by the chemical shift at δ = 2.16. A well-known method of protecting the vinyl group from radical reactions is the addition of stabilizers such as 2,2-diphenyl-1-picrylhydrazyl. However, such stabilizers were avoided in this work where we aimed to use a greener chemistry procedure to develop a more purified material for biological purposes.

### 2.2. Preparation of PBMAP Hydrogel

Hydrogels were prepared by adding PBMAP to a methacrylic acid solution and exposing the hydrogel to UV light (Figure 1a). This cross-linking process was carried out without the use of cross-linker substance. The inter-chain cross-links were responsible for keeping the material as a gel. The PBMAP solution was radiated by UV light for 1–15 min in the presence of a 20% (w/w) methacrylic acid solution. Macroscopically, no obvious hydrogel forming was observed within 3 min. The turbid solution appeared to be stratified at 5 min. After 15 min, the cross-linking reaction was complete and a light yellow hydrogel was obtained. 

### 2.3. Physical Properties of PBMAP Hydrogel

**Figure 3:**
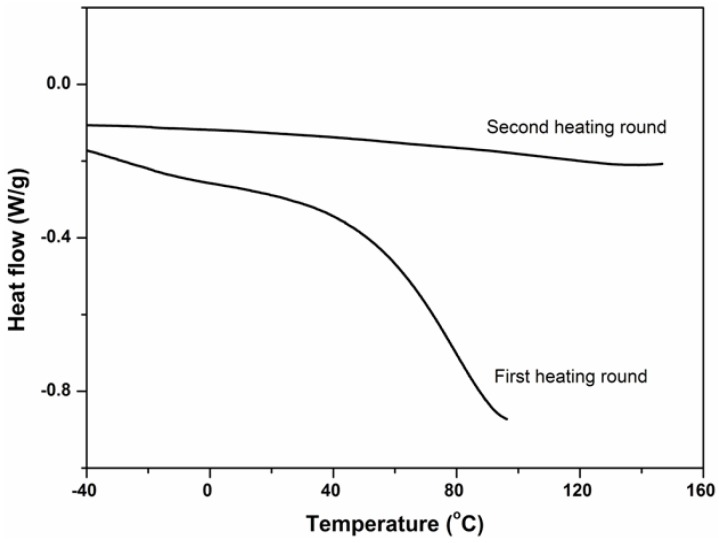
DSC curve of PBMAP hydrogel.

Thermal characterization of the hydrogel was performed with DSC, as shown in Figure 3. Two heating rounds were conducted. The hydrogel easily absorbed water when exposed to air because of the presence of carboxyl groups. During the first heating round, water in the hydrogel absorbed heat and was vaporized. In the second round, no obvious endothermic phenomenon was observed and the hydrogel showed good thermal stability; no degradation or cleavage appeared when the hydrogel was heated even up to a temperature of 150 °C. Moreover, the hydrogel maintained a similar thermal performance at temperatures above 100 °C, verifying that vinyl groups in PBMAP were completely cross-linked. 

The hydrogel was weighed before and after immersing in ultra pure water with excess water carefully removed. The swelling degree was calculated by the equation:

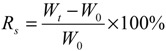
(1)
where *W_t_* and *W_0_* are the weights of the swollen and dry hydrogels, respectively. 

**Figure 4:**
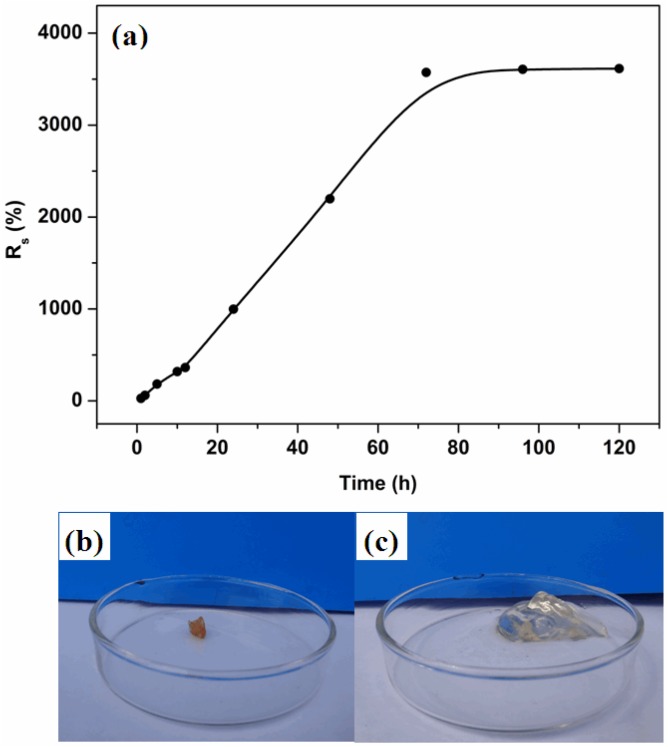
(**a**) Water uptake of the PBMAP hydrogel as a function of time. (**b**) PBMAP hydrogel in dry state. (**c**) PBMAP hydrogel swollen 35 times its own weight.

In Figure 4a, the water uptake of the hydrogel indicated remarkable absorbing ability. The absorption occurred for approximately 72 h, after which, the hydrogel had gained weight to 35 times that of the dry material. Figure 4b,c show PBMAP hydrogel in the dry and swollen states, respectively. According to the literature, the most absorbing polymer gels are derived from backbones containing ionic groups such as methacrylic acid. In fact, the structure of PBMAP hydrogel presented a synergetic absorption behavior by the poly(methacrylic acid) branches and the highly flexible backbone of the polyphosphazene. Moreover, the cross-linked network allowed for hydrogel expansion, which facilitated the diffusion of water to the hydrogel.

**Figure 5:**
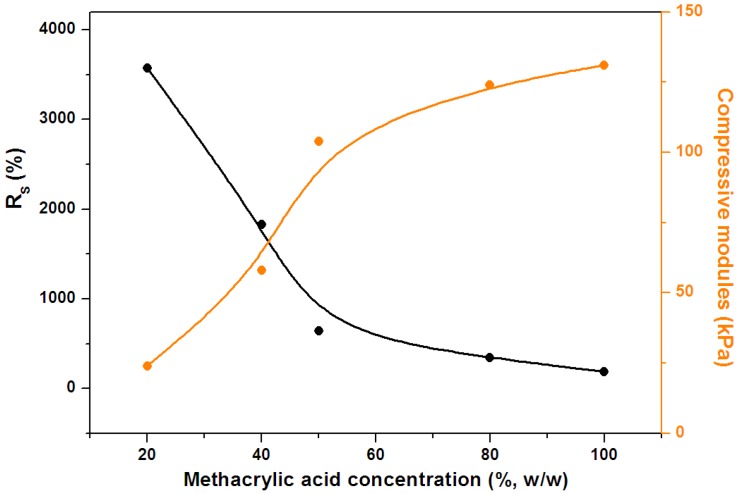
Effect of methacrylic acid concentration on hydrogel swelling ability and compressive modules.

We also studied the effect of methacrylic acid concentration on the hydrogel swelling ability. When the methacrylic acid concentration was lower than 20% (w/w), the hydrogel was weak and could be easily broken; the concentration was thus kept above 20%. All the samples tested here were placed in ultra pure water until saturated. The results in Figure 5 show that maximum water uptake was achieved at a methacrylic acid concentration of 20% (w/w). At this concentration, the lengths of branches and the cross-linking density were optimized. Further increasing the concentration increased the cross-linking density and decreased the absorption ability. Figure 5 also shows that the compressive modules increase with the increase of the methacrylic acid concentration.

### 2.4. Effect of Initial Lipase Concentration on Enzyme Loading

**Figure 6:**
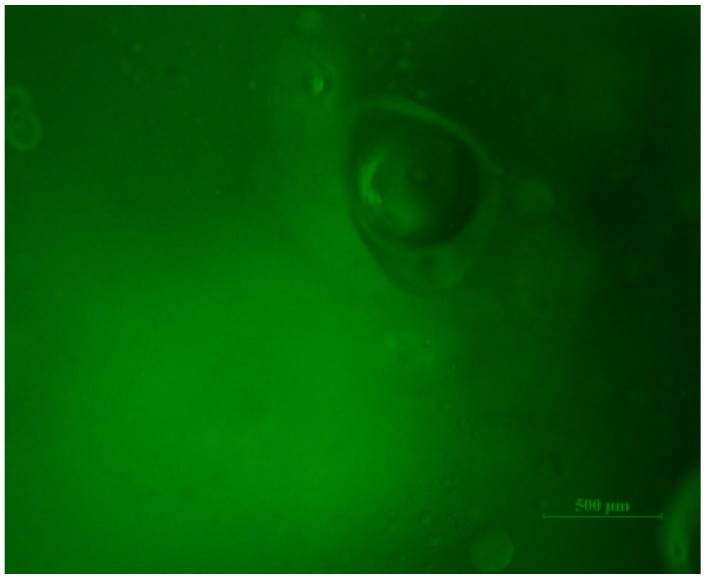
Fluorescence microscope image of polyphosphazene hydrogel immobilized with FITC-BSA (the scale bar is 500 μm).

**Figure 7:**
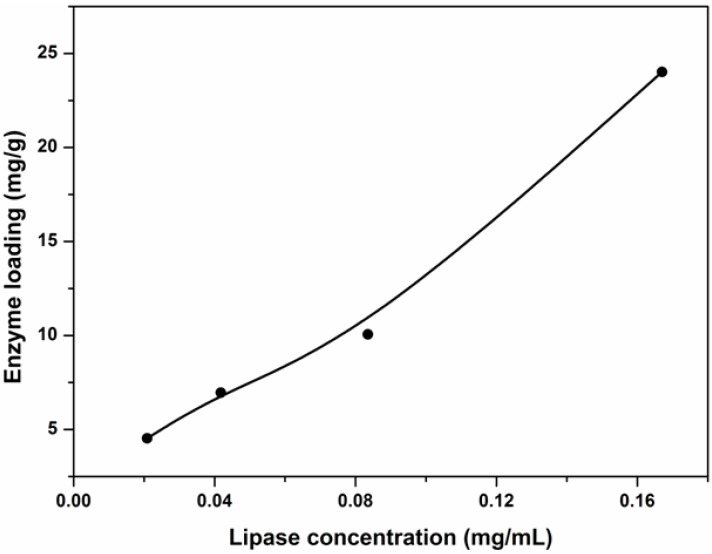
Effect of initial lipase concentration on amount of lipase absorbed in PBMAP hydrogel.

In the entrapment method, lipase entered the network of the hydrogel through diffusion. In order to verify that the proteins were entrapped within the hydrogel, we used the FITC-labeled bovine serum albumin (BSA) as a model protein for the same immobilization study. Then the BSA-immobilized hydrogel was cut and the cross-section was observed under an emission wavelength of 488 nm with a fluorescence microscope (Nikon Ti-U). The result was shown in Figure 6. According to this figure, the proteins were successfully entrapped into the hydrogel. Enzyme loading reflects the interactions between enzymes and substrates. Figure 7 shows the amount of lipase entrapped under different lipase concentrations as indicated by the enzyme loading. The hydrogel used here was prepared in the presence of a 20% (w/w) methacrylic acid solution. According to this figure, the enzyme loading increased with increasing lipase concentration and reached a maximum of 24.02 mg/g, indicating that the hydrogel owned a high capacity for biomolecules. 

### 2.5. Effect of Methacrylic Acid Concentration on Enzyme Loading and Activity

**Figure 8:**
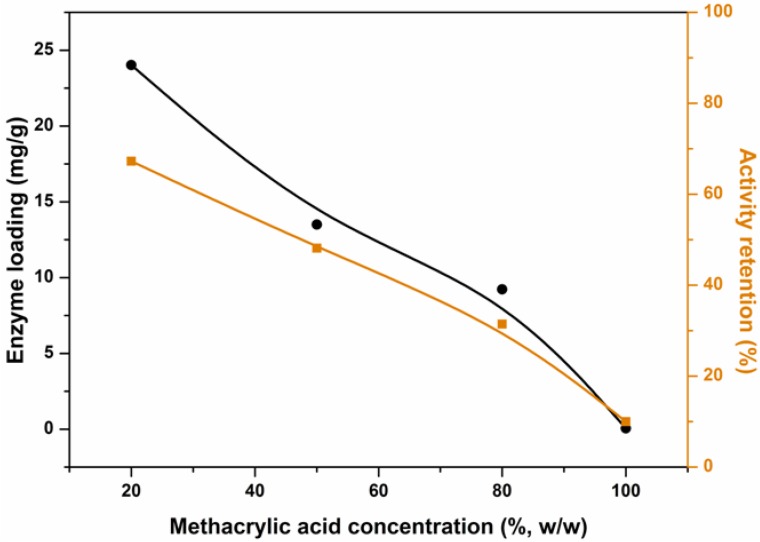
Effect of methacrylic acid concentration on lipase loading (●) and activity (■).

The lipase-immobilized hydrogel system has its advantages. The immobilized lipases retain the ability to catalyze a wide range of reactions such as alcoholysis, hydrolysis, trans-esterifications, aminolysis and enantiomer resolution. The immobilization technique also offers better catalytic stability, feasible catalyst recycling, significant operational cost reduction and simplified product purification in practical applications. The network structure of the hydrogel is vital for enzyme immobilization, and from above, we have found that the methacrylic acid concentration can affect the cross-linking degree of the hydrogel. Consequently, the lipase entrapment efficiency at a range of methacrylic acid concentrations was tested. The results are shown in Figure 8. 

Enzyme loading decreased with the increase of methacrylic acid concentration. This is due to an increase in the cross-linking density in the hydrogel; in this situation, diffusion of lipases into the hydrogel is difficult. The activity retention of the immobilized lipases decreased with an increase in methacrylic acid concentration, which was also due to the greater cross-linking degree: the denser the network structure, the greater the mass transfer limitation. According to this figure, when the concentration of methacrylic acid solution was 20% (w/w) and the enzyme loading reached a maximum of 24.02 mg/g, the activity retention was 67.25%.

### 2.6. Reuse Stability of Immobilized Lipase

**Figure 9:**
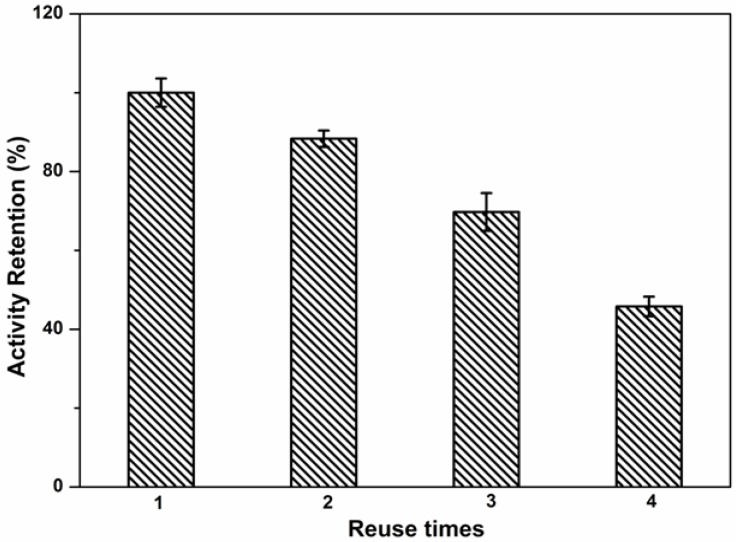
Reuse stability of immobilized lipases.

Reuse stability of the immobilized lipase is important for ensuring the economical use of the enzyme in repeated batch or continuous reactions. If the immobilized lipase has a relatively long lifetime, the cost will be remarkably decreased and its industrial implementation will be accelerated. In the reusability study, the activity of immobilized lipases in subsequent batch cycle reactions was compared with the activity in the first cycle. A slight decrease in activity appeared after the second usage. After four cycles of batch operation, less than 50% of the original activity remained (Figure 9). 

The activity loss was partially related to the inactivation of the lipase by continuous use. However, it was mostly due to the loss of entrapped lipases, which was due to the relative loose physical interaction of lipases with the hydrogel. Because this polyphosphazene hydrogel presented high absorbing ability, it can be dried and immersed in fresh lipase solution for a new round of immobilization. Moreover, the enzyme leakage can be overcome by different chemical immobilization procedures. For example, we can covalently immobilize the lipase into the hydrogel via an *N*-(3-dimethylaminopropyl)-*N'*-ethylcarbodiimide hydrochloride/*N*-hydroxysuccinimide activation process [6]. The benefit of the polyphosphazene matrix lies in its synthetic flexibility, and in this way the design of many variations of the polymer is possible; as a result, we can introduce glyoxyl, epoxyl and glutaraldehyde groups to the polymer for chemical binding with the lysine residues of the lipases [39,40,41]. Also, the lipases can be cross-linked to decrease enzyme leakage [42]. Apart from producing a covalent linkage, other methods such as chemical or physical modification of the enzymes with large polymers and usage of pre-immobilized enzymes are also possible to improve the enzyme stabilities [43,44,45,46].

## 3. Experimental

### 3.1. Materials

Hexachlorocyclotriphosphazene (HCCP, Boyuan New Material & Technology Co. Ltd., Ningbo, China) was purified by recrystallization from heptane at a temperature of 60 °C, followed by two cycles of vacuum sublimation. Poly(dichlorophosphazene) (PDCP) was synthesized via thermally initiated ring-opening polymerization of HCCP in a sealed Pyrex tube at 250 °C [38]. Tetrahydrofuran (THF) was dried by refluxing over a Na/K alloy and distilling under nitrogen. Sodium methacrylate and methacrylic acid were purchased from Sinopharm Chemical Reagent Co. Ltd. (Shanghai, China) and used without purification. Heptane was also purchased from Sinopharm Chemical Reagent Co. Ltd. and molecular sieves (4 Ǻ) were added to the heptane 24 h before use. Lipase (from *Candida rugosa*) powder (1150 units/mg solid), Bradford reagent and bovine serum albumin (BSA, molecular mass 67,000 Da) were obtained from Sigma-Aldrich Chemical Co. (St. Louis, MO, USA) and used as received. All other chemicals were of analytical grade and used without further purification.

### 3.2. Synthesis and Analysis of PBMAP

The synthesized PDCP (2.00 g, 17.3 mmol) was rinsed with petroleum ether to remove any remaining monomers and oligomers, and then dissolved in dry THF under nitrogen protection. In a rounded flask, 7.52 g (69.6 mmol) of sodium methacrylate was dissolved in 50 mL of dry THF. PDCP solution was added dropwise to the stirring sodium methacrylate suspension. The mixture was protected from light and stirred at ~35 to 40 °C for 48 h. A 50% excess of sodium methacrylate was used to assure total chlorine substitution. The reacted solution was then added dropwise to an excess amount of ethyl acetate-water (1:1 volume ratio) to precipitate the polymer product. The product was purified by washing with heptane-water (1:1 volume ratio) followed by ethyl acetate-water. After evaporation of the solvent, the material collected was a white and brittle solid. This solid could be partially dissolved in water and the insoluble part was composed of gel.

The chemical structure of PBMAP was characterized by FT-IR and ^1^H-NMR. The FT-IR spectrum of the polymer in KBr was obtained on a Brucker Vector 22 FT-IR spectrometer, while the ^1^H-NMR spectra of the sample solution in D_2_O were recorded on a Brucker Advance DMX500 nuclear magnetic resonance spectrometer.

### 3.3. PBMAP Hydrogel Formation

The hydrogel was visually detected during the purification steps. Its formation was due to the spontaneous cross-linking of the material during the reaction and when exposed to the atmosphere. This spontaneously cross-linked hydrogel was very soft and easy to break. In order to obtain hydrogels with improved mechanical properties, methacrylic acid was used for a further cross-linking process. PBMAP was added to a methacrylic acid-water solution and 2,2-dimethoxy-2-phenylacetophenone (0.05 equiv. with respect to the double bonds) was added as the photoinitiator. The system was irradiated with UV radiation (λ_max_ = 365 nm, 0.6 mW/cm^2^) for 15 min for hydrogel formation.

For the differential scanning calorimetry (DSC) test, the sample (~8 mg) was placed in an aluminum pan under a nitrogen atmosphere and heated in the first scan to 96 °C at a heating rate of 10 °C/min followed by cooling to −40 °C. A second scan was carried out by heating to 150 °C at the same heating rate used previously. The second heating run was used to observe the thermal events.

### 3.4. Swelling Behavior and Mechanical Property Measurements

Before measurements, samples were dried in a vacuum at room temperature for 24 h. The dry samples were then weighed and placed in tubes containing ultra pure water. After the swelling process, samples were collected using a spatula and transferred to a Petri dish where excess water was carefully removed using strips of filter paper. After this process, hydrogels were again weighed. The compressive stress-strain measurements were performed on hydrogels by using a CHT 4000 electronic universal testing machine (SANS, Shenzhen, China) at a velocity of 0.1 mm/min. The cylindrical gel samples were 15 mm in diameter and 10 mm in thickness.

### 3.5. Lipase Entrapment

The entrapment of lipase was performed by immersing hydrogels in lipase solution and allowing them to swell until equilibrium. Lipase solution was prepared by dissolving lipase powder in phosphate buffer solution (PBS, 0.05 M, pH 7.0). The immobilization was performed at ambient temperature. After reaching equilibrium, the samples were withdrawn and washed thoroughly with plenty of PBS (0.05 M, pH 7.0) until no free lipase was detected in the washing solution. The enzyme loading was determined using Coomassie brilliant blue reagent, following Bradford’s method [47]. BSA was used as a standard to construct a calibration curve. The entrapment capacity of lipase in the hydrogel was calculated from the protein mass balance among the initial and final lipase solutions and washings. The enzyme loading was defined as the amount of protein (milligram) per gram of hydrogel. Each reported value was the mean of at least three experiments, and the standard deviation was within *ca.* ± 5%.

### 3.6. Assay of Lipase Activity

The activity of the immobilized lipase in an aqueous medium was determined using a previously reported method [48]. Briefly, the reaction was initiated by immersing an immobilized lipase preparation in a reaction mixture composed of 1.0 mL ethanol solution containing 14.4 mM *p*-nitrophenyl palmitate and 1.0 mL PBS (0.05 M, pH 7.0). The mixture was then incubated at 25 °C under reciprocal agitation. After 5 min, the reaction was terminated by adding 2.0 mL of 0.5 M Na_2_CO_3_, followed by centrifugation for 10 min (10,000 rpm). A 0.50 mL aliquot of the supernatant was diluted 10-fold with deionized water and measured at 410 nm in a UV-Vis spectrophotometer (UV-2450, Shimadzu, Japan) against a blank without enzyme that had been treated in parallel.

One enzyme unit is defined as the amount of biocatalyst liberating 1.0 µmol *p*-nitrophenol min^−1^ for the above conditions. The activity retention value is the ratio of the specific activities of immobilized and free lipase. Each data value shown was the average of at least three parallel experiments, and the standard deviation was within *ca.* ± 5%.

## 4. Conclusions

In this work, PBMAP hydrogels were successfully prepared and lipase from *Candida rugosa* was immobilized in the hydrogel by entrapment. The hydrogel showed high absorbing ability and withstood temperatures up to 150 °C. The influence of cross-linking degree on immobilization efficiency was studied. Results showed that enzyme loading was as high as 24.02 mg/g with an activity retention of 67.25% when the methacrylic acid concentration was 20% (w/w). This work broadens the biological applications of polyphosphazenes, and based on this pathway, an efficient enzyme immobilization system can be conveniently fabricated.

## References

[1] Lee S.Y., Lee J., Chang J.H., Lee J.H. (2011). Inorganic nanomaterial-based biocatalysts. BMB Rep..

[2] Garcia-Urdiales E., Rios-Lombardia N., Mangas-Sanchez J., Gotor-Fernandez V., Gotor V. (2009). Influence of the nucleophile on the Candida antarctica lipase B-catalysed resolution of a chiral acyl donor. Chembiochem.

[3] Chen P.C., Huang X.J., Xu Z.K. (2014). Utilization of a biphasic oil/aqueous cellulose nanofiber membrane bioreactor with immobilized lipase for continuous hydrolysis of olive oil. Cellulose.

[4] Ragupathy L., Pluhar B., Ziener U., Keller H., Dyllick-Brenzinger R., Landfester K. (2010). Enzymatic aminolysis of lactones in aqueous miniemulsion: Catalysis through a novel pathway. J. Mol. Catal. B Enzym..

[5] Noureddini. H., Gao X., Philkana R.S. (2005). Immobilized Pseudomonas cepacia lipase for biodiesel fuel production from soybean oil. Bioresour. Technol..

[6] Chen P.C., Huang X.J., Xu Z.K. (2014). Kinetics-bolstered catalytic study of a high performance lipase-immobilized nanofiber membrane reactor. RSC Adv..

[7] Alves J.S., Vieira N.S., Cunha A.S., Silva A.M., Ayub M.A.Z., Fernandez-Lafuente R., Rodriques R.C. (2014). Combi-lipase for heterogeneous substrates: A new approach for hydrolysis of soybean oil using mixtures of biocatalysts. RSC Adv..

[8] Ruiz M., Galvis M., Barbosa O., Ortiz C., Torres R., Fernandez-Lafuente R. (2013). Solid-phase modification with succinic polyethyleneglycol of aminated lipase B from Candida antarctica: Effect of the immobilization protocol on enzyme catalytic properties. J. Mol. Catal. B Enzym..

[9] Hwang E.T., Gu M.B. (2013). Enzyme stabilization by nano/microsized hybrid materials. Eng. Life Sci..

[10] Lee C.H., Lin T.S., Mou C.Y. (2009). Mesoporous materials for encapsulating enzymes. Nano Today.

[11] Garcia-Galan C., Berenguer-Murcia A., Fernandez-Lafuente R., Rodriques R.C. (2011). Potential of different enzyme immobilization strategies to improve enzyme performance. Adv. Synth. Catal..

[12] Rodrigues R.C., Bolivar J.M., Volpato G., Filice M., Fernandez-Lafuente R., Guisan J.M. (2009). Improved reactivation of immobilized-stabilized lipase from Thermomyces lanuginosus by its coating with highly hydrophilic polymers. J. Biotechnol..

[13] Kobayashi S., Kobayashi J., Mori Y. (2006). Novel immobilization method of enzymes using a hydrophilic polymer support. Chem. Commun..

[14] Iyer P.V., Ananthanarayan L. (2008). Enzyme stability and stabilization – Aqueous and non-aqueous environment. Process Biochem..

[15] Coradin T., Nassif N., Livage J. (2003). Silica-alginate composites for microencapsulation. Appl. Microbiol. Biotechnol..

[16] Mateo C., Palomo J.M., Fernandez-Lorente G., Guisan J.M., Fernandez-Lafuente R. (2007). Improvement of enzyme activity, stability and selectivity via immobilization techniques. Enzyme Microb. Technol..

[17] Caruso F., Wang Y.J. (2004). Enzyme encapsulation in nanoporous silica spherest. Chem. Commun..

[18] Rodrigues R.C., Berenguer-Murcia A., Fernandez-Lafuente R. (2011). Coupling chemical modification and immobilization to improve the catalytic performance of enzymes. Adv. Synth. Catal..

[19] Adlercreutz P. (2013). Immobilisation and application of lipase in organic media. Chem. Soc. Rev..

[20] Tiller J.C., Bruns N. (2005). Amphiphilic network as nanoreactorfor enzymes in organic solvents. Nano Lett..

[21] Peppas L.B., Peppas N.A. (1989). Solute and penetrants diffusion in swellable polymers. IX. The mechanisms of drug release from pH-sensitive swelling controlled systems. J. Control. Release.

[22] Nykänen V.P.S., Nykänen A., Puska M.A., Silva G.G., Ruokolainen J. (2011). Dual-responsive and super absorbing thermally cross-linked hydrogel based on methacrylate substituted polyphosphazene. Soft Matter.

[23] Gawlitza K., Georgieva R., Tavraz N., Keller J., von Klitzing R. (2013). Immobilization of water-soluble HRP within poly-N-isopropylacrylamide microgel particles for use in organic media. Langmuir.

[24] Grunwald P., Hansen K., Gunßer K. (1997). The determination of effective diffusion coefficients in a polysaccharide matrix used for the immobilization of biocatalysts. Solid State Ionics.

[25] Mansson R., Frenning G., Malmsten M. (2013). Factors affecting enzymatic degradation of microgel-bound peptides. Biomacromolecules.

[26] Lei J., Fan J., Yu C., Zhang L., Jiang S., Tu B., Zhao D. (2004). Immobilization of enzymes in mesoporous materials: Controlling the entrance to nanospace. Micropor. Mesopor. Mat..

[27] Chen H., Liu L.H., Wang L.S., Ching C.B., Yu H.W., Yang Y.Y. (2008). Thermally responsive reversed micelles for immobilization of enzymes. Adv. Funct. Mater..

[28] Xu J., Zhao Q.H., Jin Y.M., Qiu L.Y. (2014). High loading of hydrophilic/hydrophobic doxorubicin into polyphosphazene polymersome for breast cancer therapy. Nanomed.Nanotech. Biol. Med..

[29] Allcock H.R., Kwon S. (1989). An ionically cross-Linkable polyphosphazene: Poly [bis(carboxylatophenoxy)phosphazene] and its hydrogels and membranes. Macromolecules.

[30] Allcock H.R., Pucher S.R., Turner M.L., Fitzpatrick R.J. (1992). Poly(organophosphazenes) with poly(alky1 ether) side groups: A study of their water solubility and the swelling characteristics of their hydrogels. Macromolecules.

[31] Allcock H.R., Kwon S., Riding G.H., Fitzpatrick R.J., Bennett J.L. (1988). Hydrophilic polyphosphazenes as hydrogels: Radiation cross-linking and hydrogel characteristics of poly [bis(methoxyethoxyethoxy)phosphazene]. Biomaterials.

[32] Allcock H.R., Pucher S.R., Visscher K.B. (1994). The activity of urea amidohydrolase immobilized within poly[di(methoxyethoxyethoxy)phosphazene] hydrogels. Biomaterials.

[33] Allcock H.R., Ambrosio A.M.A. (1996). Synthesis and characterization of pH-sensitive poly(organophosphazene) hydrogel. Biomaterials.

[34] Allcock H.R., Chang Y.K. (2003). A covalently interconnected phosphazene-silicate hybrid network: Synthesis, characterization, and hydrogel diffusion-related application. Adv. Mater..

[35] Tian Z.C., Chen C., Allcock H.R. (2013). Injectable and biodegradable supramolecular hydrogels by inclusion complexation between poly(organophosphazenes) and α-cyclodextrin. Macromolecules.

[36] Qian Y.C., Huang X.J., Chen C., Ren N., Huang X., Xu Z.K. (2012). A versatile approach to the synthesis of polyphosphazene derivatives via the thiol-ene reaction. J. Polym. Sci. Pol. Chem..

[37] Xue L.W., Mao L.X., Cai Q., Yang X.P., Jin R.G. (2011). Preparation of amino acid ester substituted polyphosphazene microparticles via electrohydrodynamic atomization. Polym. Adv. Technol..

[38] Qian Y.C., Ren N., Huang X.J., Chen C., Yu A.G., Xu Z.K. (2013). Glycosylation of polyphosphazene nanofibrous membrane by click chemistry for protein recognition. Macromol. Chem. Phys..

[39] Mateo C., Palomo J.M., Fuentes M., Betancor L., Grazu V., Lopez-Gallego F., Pessela B.C.C., Hidalgo A., Fernandez-Lorente G., Fernandez-Lafuente R. (2006). Glyoxyl agarose: A fully inert and hydrophilic support for immobilization and high stabilization of proteins. Enzyme Microb. Technol..

[40] Mateo C., Grazu V., Pessela B.C.C., Montes T., Palomo J.M., Torres R., Lopez-Gallego F., Fernandez-Lafuente R., Guisan J.M. (2007). Advances in the design of new epoxy supports for enzyme immobilization-stabilization. Biochem. Soc. Trans..

[41] Betancor L., Lopez-Gallego F., Hidalgo A., Alonso-Morales N., Dellamora-Ortiz G., Mateo C., Fernandez-Lafuente R., Guisan J.M. (2006). Different mechanisms of protein immobilization on glutaraldehyde activated supports: Effect of support activation and immobilization conditions. Enzyme Microb. Technol..

[42] Cao L., van Langen L., Sheldon R.A. (2003). Immobilized enzymes: Carrier-bound or carrier-free?. Curr. Opin. Biotechnol..

[43] Chalker J.M., Bernardes G.J.L., Lin Y.A., Davis B.G. (2009). Chemical modification of proteins at cysteine: Opportunities in chemistry and biology. Chem. Asian J..

[44] Fernandez-Lafuente R., Rosell C.M., Caanan-Haden L., Rodes L., Guisan J.M. (1999). Facile synthesis of artificial enzyme nano-environments via solid-phase chemistry of immobilized derivatives: Dramatic stabilization of penicillin acylase *versus* organic solvents. Enzyme Microb. Technol..

[45] Fuentes M., Segura R.L., Abian O., Betancor L., Hidalgo A., Mateo C., Fernandez-Lafuente R., Guisan J.M. (2004). Determination of protein-protein interactions through aldehyde-dextran intermolecular cross-linking. Proteomics.

[46] Wilson L., Fernandez-Lorente G., Fernandez-Lafuente R., Illanes A., Guisan J.M., Palomo J.M. (2005). CLEAs of lipases and poly-ionic polymers: A simple way of preparing stable biocatalysts with improved properties. Enzyme Microb. Technol..

[47] Bradford M. (1976). A rapid and sensitive method for the quantition of microgram quantities of protein utilizing the principle of dyebinding. Anal. Biochem..

[48] Huang X.J., Chen P.C., Huang F., Ou Y., Chen M.R., Xu Z.K. (2011). Immobilization of Candida rugosa lipase on electrospun cellulose nanofiber membrane. J. Mol. Catal. B Enzym..

